# Insulin resistance and metabonomics analysis of fatty liver haemorrhagic syndrome in laying hens induced by a high-energy low-protein diet

**DOI:** 10.1038/s41598-019-46183-y

**Published:** 2019-07-12

**Authors:** Yu Zhuang, Chenghong Xing, Huabin Cao, Caiying Zhang, Junrong Luo, Xiaoquan Guo, Guoliang Hu

**Affiliations:** Jiangxi Provincial Key Laboratory for Animal Health, Institute of Animal Population Health, College of Animal Science and Technology, Jiangxi Agricultural University, No. 1101 Zhimin Avenue, Economic and Technological Development District, Nanchang, 330045 Jiangxi P.R. China

**Keywords:** Animal physiology, Metabolomics

## Abstract

Fatty liver haemorrhagic syndrome (FLHS) is a widespread metabolic disease in laying hens that causes a decrease in egg production and even death. Insulin resistance is a major contributor to the pathogenesis of nonalcoholic fatty liver disease. However, the relationship between FLHS and the insulin resistance mechanisms underlying FLHS is not well elucidated. Therefore, we established an FLHS model induced by feeding a high-energy low-protein diet. In the current study, we found that the fasting glucose and insulin concentrations were elevated in the FLHS group compared with the control group during the experimental period. The results of the oral glucose tolerance test (OGTT) and insulin sensitivity test (IST) showed a high level of insulin resistance in the FLHS model. InsR, 4EBP-1, Glut-1 and Glut-3 mRNA expression were decreased, and TOR, S6K1, and FOXO1 were elevated (*P* < *0.05*). Metabolomic analysis with GC/MS identified 46 differentially expressed metabolites between these two groups, and of these, 14 kinds of metabolism molecules and 32 kinds of small metabolism molecules were decreased (*P* < *0.05*). Further investigation showed that glucose, lipid and amino acid metabolism blocks in the progression of FLHS by GO functional and pathway analysis. Overall, these results suggest that insulin resistance participated in FLHS; comprehensively, metabolites participated in the dysregulated biological process.

## Introduction

Fatty liver haemorrhagic syndrome (FLHS) is a nutritional and metabolic disease characterized by a lipid metabolism disorder that is harmful to the development of the layer breeding industry^1–3^. Due to the adoption of caged feeding technology and advances in breeding in the layer industry, FLHS, which is commonly observed, has periods of latency and sudden outbreaks, causing a sudden drop in egg production. This syndrome has been reported to be the most common non-infectious cause of high mortality in layers^2^. An epidemiological survey showed that nutrition, genetics, and environmental toxins are highly associated with FLHS outbreaks on layer farms. A recent study reported that a low-protein high-energy (HELP) diet is the main cause of FLHS occurrence^4^.

FLHS is characterized by excessive ectopic fat deposition in the liver and abdominal cavity along with a haemorrhagic and fragile liver. A study by Song and his colleagues showed that abnormal expression of apoAI and apoB_100_ in the liver was observed in the FLHS model induced by a low-protein high-energy diet^5^. This result suggested that lipid transfer disorder was a component of FLHS. In addition, studies have shown a robust increase in de novo lipogenesis and inhibition of the oxidation of free fatty acids in various FLHS models. Jiang *et al*. reported the syndrome in laying hens with upregulated bone turnover and exacerbated skeletal damage along with increased oestrogen concentration^6^. Overall, these pathological observations in FLHS concur with the characteristics of nonalcoholic fatty liver disease (NAFLD): ①fatty over-synthesis, ②fatty transportation disturbance, and ③fatty acid oxidation blocking^7–9^. However, the aetiology and pathogenesis of FLHS have not been fully elucidated due to its complex inducement on layer farms.

Insulin is the most potent anabolic hormone known and essential for appropriate tissue development, growth, and regulation of most metabolism^10^. Insulin binding with insulin receptor (InsR) activates a cascade of molecules collectively known as the PI3K/Akt/TOR signalling pathway, which is responsible for most metabolic actions of insulin^11^. Multiple factors can block insulin signalling, ultimately resulting in hepatic triglyceride accumulation and impaired glucose homeostasis. Recent studies have shown that insulin resistance is highly associated with NAFLD^12,13^. However, no reports have elaborated on the relationship between insulin resistance and the occurrence and development of FLHS.

Previous studies revealed that glucose homeostasis and lipid metabolism in chickens presented several peculiarities^14–16^. Chickens and other avians have two- to four fold higher glucose concentrations than mammals with similar body masses, as well as a natural “apparent insulin refractoriness^17,18^”. Another peculiarity is that lipogenesis in chickens occurs essentially in the liver, with low activity of lipogenic enzymes in avian adipose tissue and lacking vital glucose transport protein 4 (GLUT4)^19^. Herein, avian species are liable to form hyperglycaemia and hyperlipaemia, which might have toxic effects on the liver. Therefore, we hypothesize that the particularities of insulin and glucose metabolism in avians play an important role in the progression of FLHS. In the current study, we first established the FLHS model by feeding layers a high-energy low-protein (HELP) diet, and then, glucose, insulin and insulin resistance and expression of the insulin signalling pathway were measured during the experimental period to analyse the relationship between the insulin resistance and FLHS. In addition, the liver metabolites from the FLHS model provide a complete understanding of the development of FLHS and a better understanding of insulin resistance in FLHS.

## Results

### Characteristics of the FLHS model induced by a HELP diet

**Figure 1:**
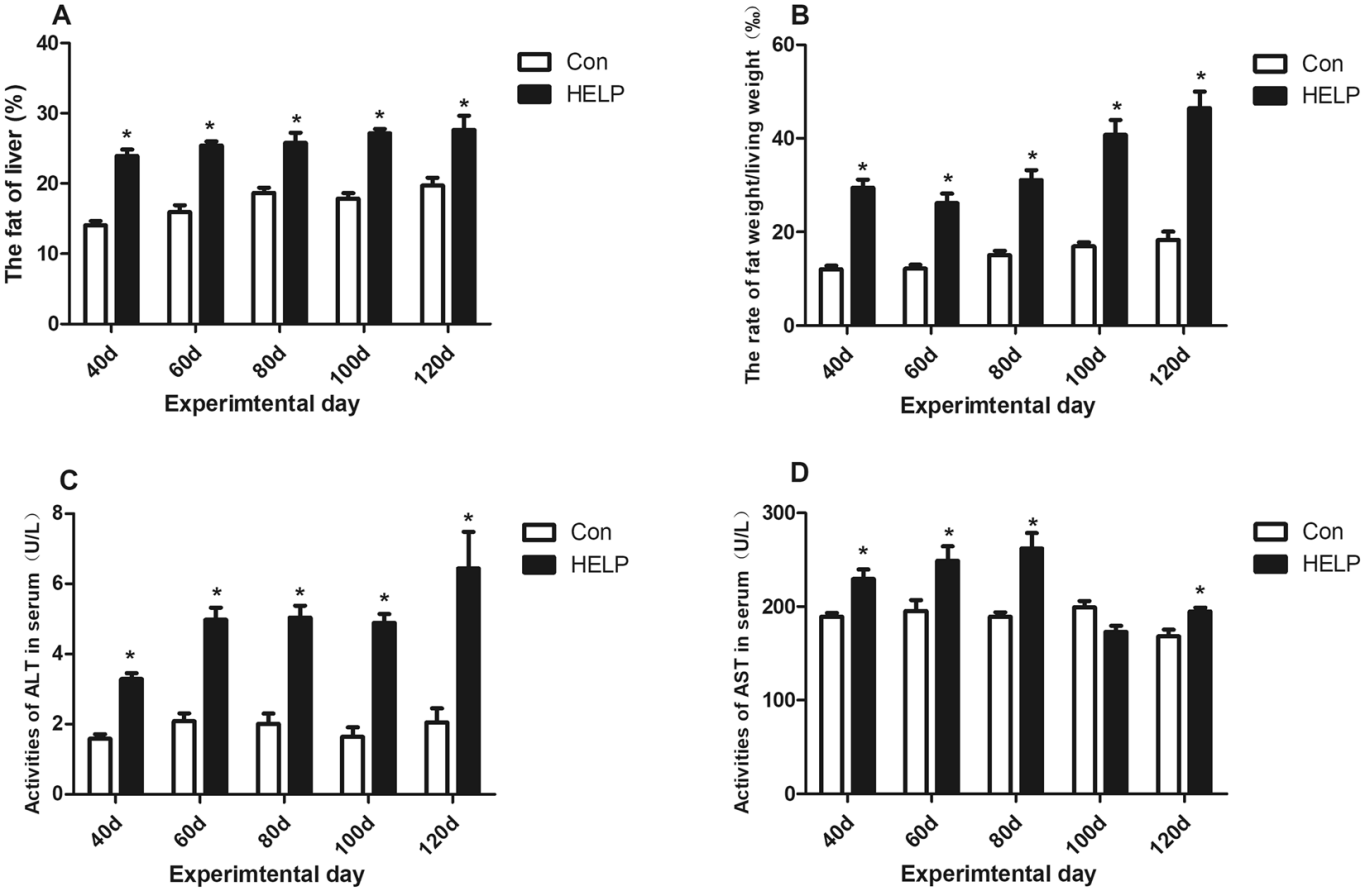
Characteristics of fatty liver hemorrhagic syndorome (FLHS) model induced by high erergy-low protein (HELP) Diet. (**A**) Rate of liver fat (%), (**B**) Rate of abdnormal fat, (**C**) Activities of ALT and (**D**) AST in serum from layer fed normal diet (control group) in layers fed normal diet and HELP diet on day 40, 80 and 120. All values are means ± SEM. Means with asterisks are significantly different from values of layer in control group.

**Figure 2:**
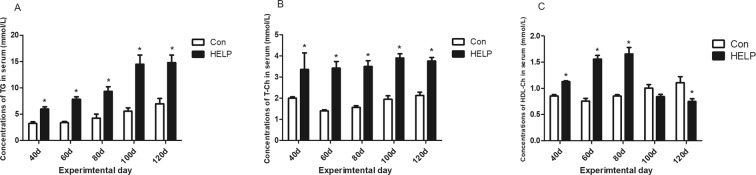
Concentration of serum biochemical induces. (**A**) Concentration of Triglyceride (TG), (**B**) T-Ch and HDL-Ch (**C**) in serum from layer fed normal diet (Con) and high energy-low protein diet (HELP) on day 40, 80 and 120. All values are means ± SEM. Means with asterisks are significantly different from values of layer in control group.

The lipid accumulation in layers is shown in Fig. 1. The liver fat and abdominal fat in the HELP group were increased (*P* < *0.05*) on experimental days 40, 80 and 120 compared with those in the control group (Fig. 1A,B). The liver indexes were relatively complicated in the HELP group compared with the control group; these indexes were significantly increased (*P* < *0.05*) on days 40 and 120 in the HELP group (Fig. S1). Furthermore, the alanine aminotransferase (ALT) and aspartate aminotransferase (AST) concentrations were significantly increased (*P* < *0.05*) in the HELP group compared with the control group on days 40, 80 and 120 (Fig. 1C,D). In addition, the serum biochemical indexes are shown in Fig. 2. Compared with those in the control group, the triglyceride (TG) and cholesterol concentrations in the HELP group were significantly elevated (*P* < *0.05*), whereas the high-density lipoprotein cholesterol (HDL-Ch) content was significantly increased in the early days and then decreased on day 120 (*P* < *0.05*).

**Figure 3:**
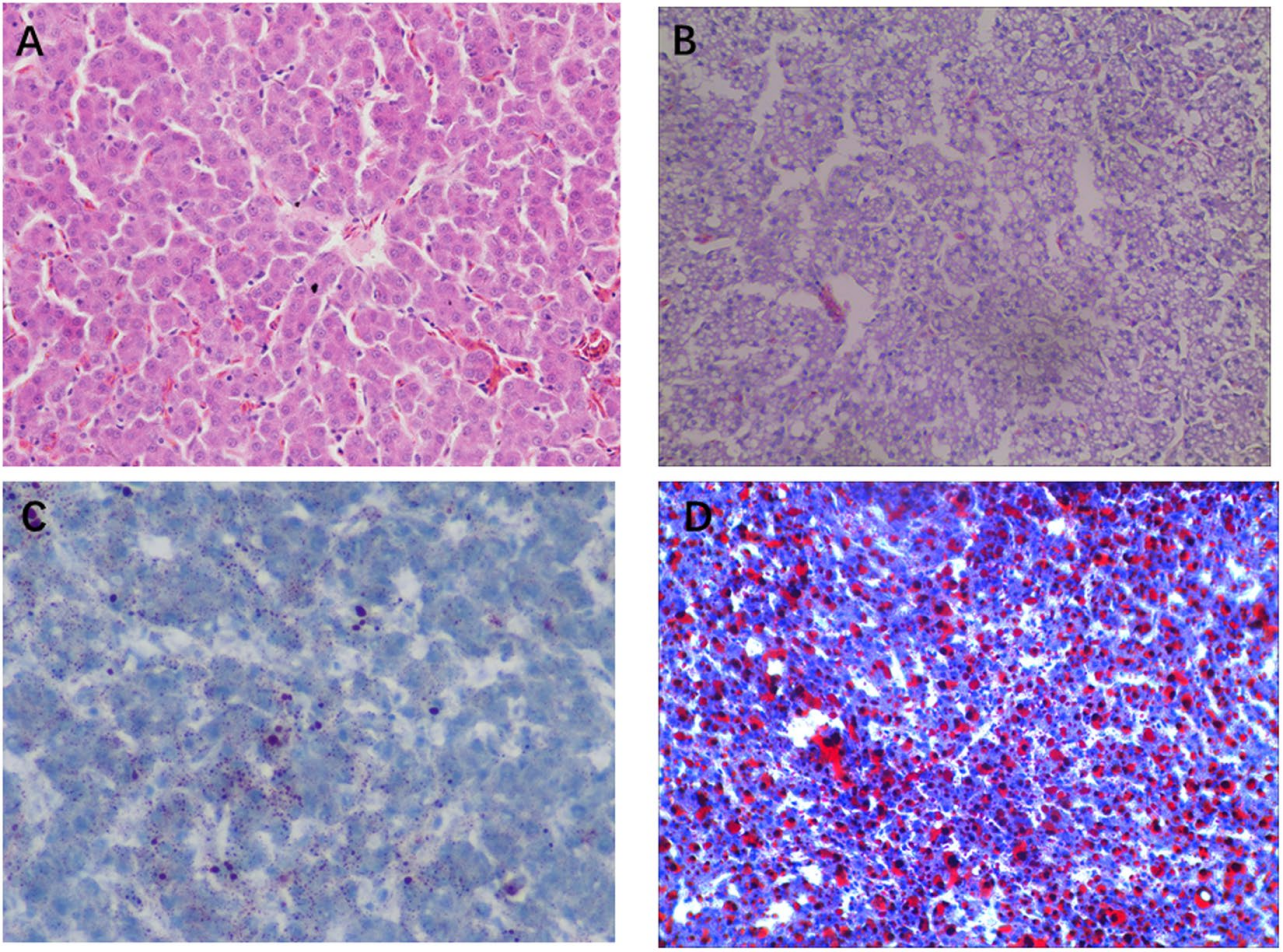
Histopathological Observation by Hematoxylin-eosin (H.E) staining and Oil red staining (X400) of the liver tissue of layer in high energy-low protein (HELP) group and control group. (**A**,**C**) Normal liver from control group on experimental days 120 were stained by H.E and Oil red, respectively. (**B**,**D**) Fat pathological changes in HELP group were stained by H.E and Oil red, respectively.

Liver histology in the control group showed a clear liver cell structure with regular morphology with HE and Oil red O staining (Fig. 3). However, in the HELP group, HE and Oil red O staining showed that liver cells presented fatty pathological changes, with abundant lipid droplets accumulating in the cytoplasm and extrusion of the nucleus to the cell edge. In addition, ultrastructural observation further found that mitochondrial cristae structures became blurred and swollen, mitochondrial abundance declined, and autophagosomes in the cytoplasm increased in the HELP group compared with the control group. Overall, steatosis and necrosis were clearly observed in layers from the HELP group (Fig. S2).

### IL-6 and TNF-α contents in serum

**Figure 4:**
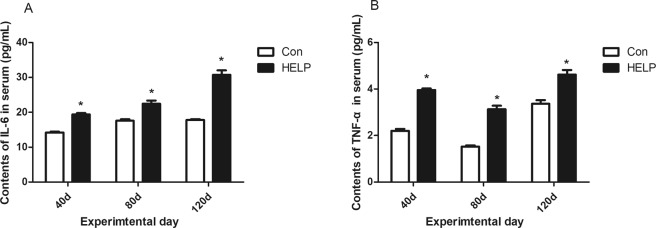
Contents of IL-6 and TNF-α in serum. (**A**) Contents of IL-6, (**B**) TNF-α in serum from layer fed normal diet (control group) and high energy-low protein (HELP) diet on days 40, 80, 120. All values are means ± SEM. Means with asterisks are significantly different from values of layer in control group.

The contents of IL-6 and TNF-α, shown in Fig. 4, were significantly elevated (*P* < *0.05*) in layers from the HELP group compared with the layers from the control group on experimental days 40, 80 and 120.

### Fasting insulin and glucose concentrations

The fasting insulin and glucose concentrations are shown in Table 1. The insulin and glucose concentrations in layers in the HELP group were significantly elevated (*P* < *0.05*) on days 40, 80 and 120 compared with those in layers in the control group.

**Table 1:** Chang of Insulin and relative index.

Index	Groups	Experimental period (Day)		
		40d	80d	120d
**Insulin (mU/L)**	Con	2.54 ± 0.44^a^	2.25 ± 0.18^a^	2.21 ± 0.06^a^
	HELP	3.26 ± 0.12^b^	2.98 ± 0.29^b^	3.63 ± 0.26^b^
**Glucose (nmol/L)**	Con	9.82 ± 1.07^a^	9.80 ± 0.66^a^	9.89 ± 0.55^a^
	HELP	10.86 ± 0.87^b^	10.37 ± 0.45^b^	11.14 ± 0.77^b^

Note: Fast insulin and glucose in serum from layer fed normal diet (Con) and high energy-low protein diet (HELP) on days 40, 80, 120 were determined by Elisa commercial kits and handheld glucometer. All values are means ± SEM. Values with same smaller letter superscripts mean no significant difference (*P* > *0.05*), with different small letter superscripts mean very significant difference (*P* > *0.05*) in the same column.

### Oral glucose tolerance test (OGTT) and insulin sensitivity test (IST)

**Figure 5:**
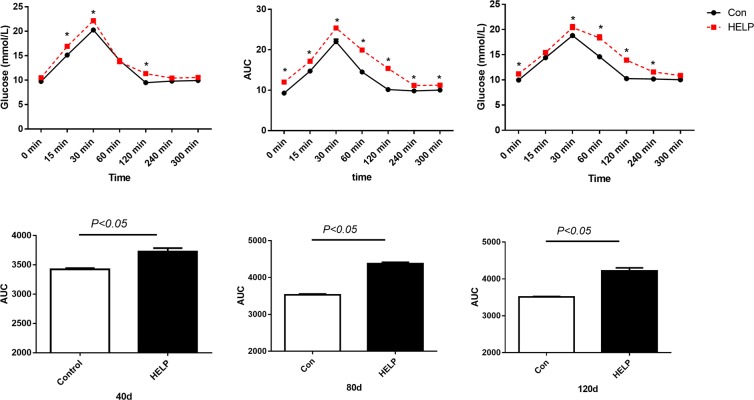
Oral glucose tolerance test (OGTT) on experimental days 40, 80 and 120 in both groups. Plasma glucose levels after an oral glucose load (2 g/kg BW, 20% w/v H_2_O) in layers from the control and HELP groups on days (**A**) 40, (**B**) 80 and (**C**) 120. Plasma glucose was measured using a handheld glucometer at the indicated time points. Area under the curve (AUC) calculated from the glucose curve at the indicated time points on days (**D**) 40, (**E**) 80 and (**F**) 120. All values are means ± SEM. Means with asterisks are significantly different from the means of the layers in the control group.

**Figure 6:**
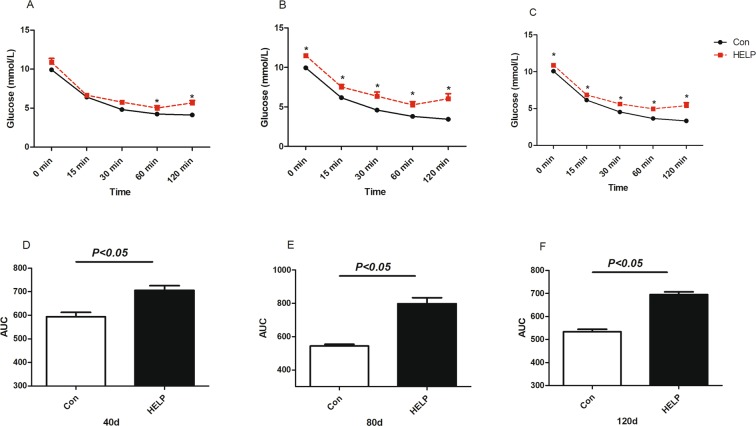
Insulin sensitivity test (IST) on experimental days 40, 80 and 120 in both groups. Plasma glucose after received 100 ug/kg BW bovine insulin in layer from control and HELP group on days (**A**) 40, (B)80, (**C**)120. Plasma glucose were measured using a handheld glucometer at the indicated time points. Area under the curve (AUC) calculated from glucose curve at the indicated time points on days 40 (**D**), 80 (**E**) and 120 (**F**). All values are means ± SEM. Means with asterisks are significantly different from values of layer in control group.

The OGTT and IST in both groups are shown in Figs 5 and 6, respectively. Plasma glucose levels were significantly elevated after oral glucose load at 30 min in layers from the HELP group (*P* < *0.05*) compared with control group during experimental period. The glycaemic curve in the control group was significantly decreased (*P* < *0.05*) compared with HELP group at the 60 min and 120 min time points on experimental days 80 and 120. In additional, the area under the curve (AUC) calculated from the glucose curve in the HELP group was significantly (*P* < *0.05*) increased compared with the AUC in the control group on experimental days. In addition, plasma glucose in layers from the HELP group was significantly increased after receiving insulin *(P* < *0.05*) compared with that in layers from the control group at indicated points on experimental days. The corresponding AUC in the HELP group was significantly increased (*P* < *0.05*) compared with that in the control group. Overall, insulin sensitivity was declined in the HELP group compared with the control group according to the OGTT and IST.

### Relative mRNA expression of insulin signalling pathway factors

**Figure 7:**
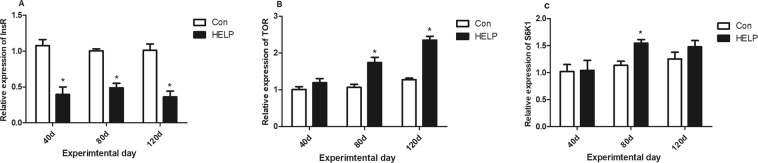
Expression of factors in insulin signal pathway. The relative expression of InsR (**A**), TOR (**B**), S6K1 (**C**) from liver in layers fed normal diet (control group) and HELP diet on days 40, 80, 120. All values are means ± SEM. Means with asterisks are significantly different from values of layer in control group. InsR: Insulin Receptor, TOR: Target Of Rapamycin, S6K1: Ribosomal S6 kinase1.

The mRNA expression levels of InsR, TOR, S6K1 and 4EBP are shown in Fig. 7. Compared with the layers in the control group, the layers in the HELP group showed a significant decrease in expression of InsR (*P* < *0.05*) on experimental days 80 and 120. The mRNA expression of TOR and S6K was significantly elevated in the HELP group compared with the control group on experimental day 80. Moreover, the mRNA expression levels of the downstream effectors of the insulin signalling pathway are shown in Fig. S3. The mRNA expression levels of Glut-1, Glut-3 and 4EBP were significantly decreased, and the mRNA expression levels of FOXO-1 and Srebp-1 were significantly elevated in the HELP group compared with the control group on experimental days 80 and 120 (*P* < *0.05*).

### Liver metabolites

**Figure 8:**
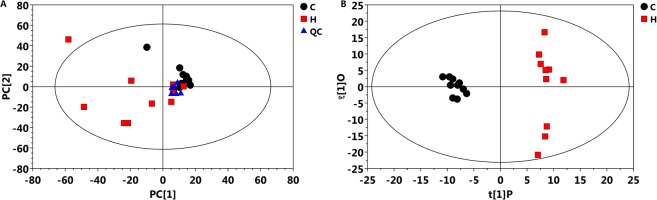
Score plot of principal component analysis (PCA) model and orthogonal partial least squares discrimination analysis (OPLS-DA) model obtained from C and H, Outlying samples of the ellipse region with the 95% confidence interval were excluded by Hotelling’s T2 test. The score plots showed a significant separation between control group and HELP group. C: control group, H: HELP group, QC: Quality Control.

The MS data of liver metabolites from the control and HELP groups on experimental day 120 were applied to the OPLS-DA score plot (Fig. 8). The PCA and OLPS-DA score plots of liver metabolites showed distinct clustering for each group of layers in the control and HELP groups. Both groups were clearly discriminated from each other by the primary component t (1) or the secondary component t (2) based on the model with R^2^X (cum) and R^2^Y (cum), indicating the goodness of fit of the data, and with Q^2^ (cum) values estimating the predictive ability of the model in PCA and OLPS-DA (Table S1). In addition, the PLS-DA models were validated by a permutation test. *R* intercept values of all models, the intercept of permutations test with R^2^ values of 0.954 and Q^2^ values of −0.04, indicated that the models were not overfitted and had good viability (Fig. S4). A loading plot of the OPLS-DA model obtained for the control and HELP groups (Fig. S5) showed that ornithine (696), maltose (1045), O-acetylserine (270), O-ethanolamine phospholipid (673) and alanine (117) were potential markers between the two groups.

### Quantitative analysis of liver metabolites

**Figure 9:**
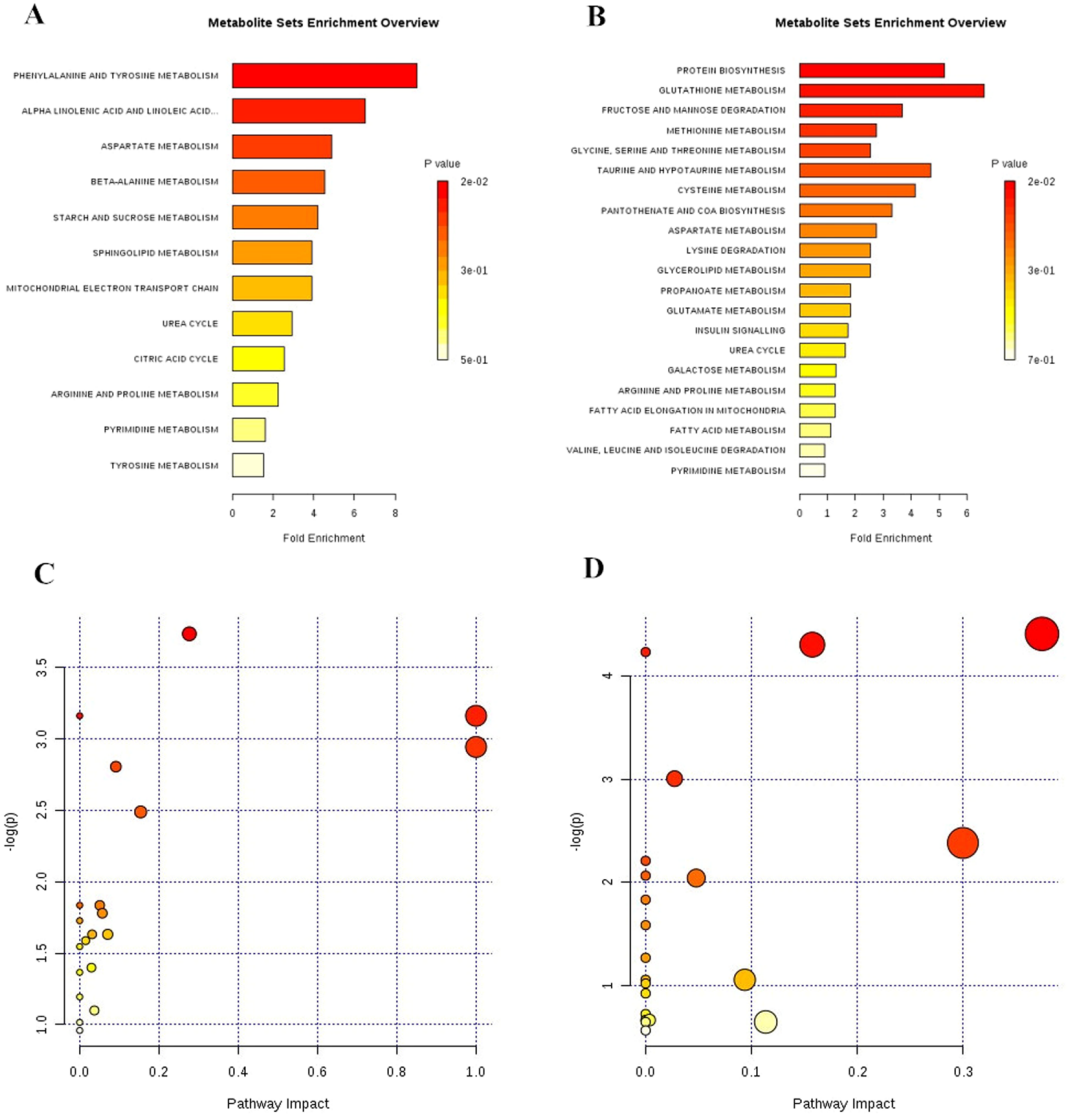
Enrichment and pathway analyses of upregulated or downregulated metabolites by GC-MS/MS in high energy-low protein (HELP) group compared with the control group. Over-representation analysis (ORA) was performed when a list of compound names is provided. In addition, ORA was implemented using the hypergeometric test to evaluate whether a particular metabolite set is represented more than expected by chance within the given compound list. One-tailed p values are provided after adjusting for multiple testing. (**A**,**B**) Present upregulation and metabolite enrichment, respectively. In the context of enrichment analysis in pathway analysis, we tested whether compounds involved in a particular pathway are enriched compared to random hits. MetPA offers one of the most commonly used methods for ORA, Fisher’s Exact test. (**C**,**D**) Present upregulation and metabolite enrichment, respectively.

The normalized intensities of whole metabolites detected by GC-MS in liver extracts were statistically analysed by a nonparametric t test. All metabolites, including 46 metabolites in the liver, were significantly affected by a high-energy low-protein diet (Table 2). Among these metabolites, 14 metabolites (fumarate, alanine, glutamic acid and O-phosphorylethanolamine, etc.) were significantly elevated, and 32 metabolites (valine, isoleucine, 5-methoxytryptamine, monostearin, etc.) were significantly decreased in the HELP group compared with the control group. In addition, the enrichment analyses^20^ showed that phenylalanine and tyrosine metabolism was significantly increased, while protein biosynthesis and glutathione metabolism were significantly decreased in the HELP group (*P* < *0.05*) compared with the control group (Fig. 9A,B). The pathway analyses^20^ showed that glutathione metabolism, cysteine and methionine metabolism, valine, leucine and isoleucine biosynthesis, fructose and mannose metabolism were significantly decreased and that alanine, aspartate and glutamate metabolism, phenylalanine, tyrosine and tryptophan biosynthesis and linoleic acid metabolism were significantly increased in the HELP group (*P* < *0.05*) compared with the control group (Fig. 9C,D).

**Table 2:** Different metabolites in Control and HELP Group.

HELP VS CON	Differentail metabolites	P value	Differentail metabolites	P value
↑	fumaric acid	0.04852288	alanine	0.008562986
	Linoleic Acid	0.008325082	glutamic acid	0.037667079
	maltose	0.000461638	hydroxylamine	2.3604E-05
	1-Hexadecanol	0.010880608	N-Carbamylglutamate	0.013374861
	hexadecane	0.01307622	O-Phosphorylethanolamine	0.001846875
	phenylpyruvate	0.048c675055	5,6-dihydrouracil	0.024502167
	Methyl jasmonate	0.017966067	O-acetylserine	0.000452172
**↓**	palmitic acid	0.029890973	valine	0.014577493
	adipic acid	0.046610053	L-Allothreonine	0.001168747
	N-ethylmaleamic acid	0.047373433	Isoleucine	0.021310303
	ascorbate	0.002771808	ornithine	0.000872746
	Monostearin	0.019313325	N-Methyl-DL-alanine	0.002435752
	sorbitol	0.008171542	L-cysteine	0.002407664
	fucose	0.005025088	Threonine	0.001409681
	Lyxose	0.039162168	5-Methoxytryptamine	0.000406018
	alpha-D-glucosamine 1-phosphate	0.007504568	cystine	0.003830458
	Tagatose 1	0.027166792	methionine	0.025251853
	Levoglucosan	0.045374919	2-hydroxypyridine	0.00435651
	1,5-Anhydroglucitol	0.020448929	3-Hydroxypyridine	0.004653341
	Dithioerythritol	0.026999158	5’-methylthioadenosine	0.002491219
	octanal	0.02149557	alpha-Aminoadipic acid	0.00042186
	5-Methylresorcinol	0.046626525	N-acetyl-L-aspartic acid 1	0.047373433
			glutathione	0.00191077
			2-Deoxyuridine	0.000188016

Note: Different metabolites in liver from layer fed normal diet (Con) and high energy-low protein diet (HELP) on days 120 were determined by GC/MS method. Means with Symbol “**↑**” were up-regulation different metabolites compared with layers from control group, while Symbol “**↓**” were down-regulation different metabolites compared with layers from control group.

## Discussion

FLHS is a widespread metabolic disease in laying hens that causes adult deaths and a sudden drop in egg production^2,5^. However, the molecular mechanisms underlying FLHS are still poorly understood. Current studies generally indicate that nutrients, hormones, environmental conditions, temperature and metabolic input contribute to the occurrence of FLHS. Therefore, an appropriate FLHS model is the basis for investigating its mechanism. An epidemiological survey of layer farms showed that a HELP diet is the primary natural cause of FLHS^1,3^. In addition, our previous studies also successfully established an FLHS model by feeding a HELP diet. Thus, we used a HELP diet (metabolic energy 12.97 MJ/kg, crude protein 12.00%) combined with caged feeding in a high-temperature/high-humidity environment to induce the FLHS model in laying hens.

In the present study, the layers consuming the HELP diet exhibited characteristics of FLHS on experimental day 80, and clinical symptoms, biochemical indexes and necropsy were consistent with those in natural cases. Histopathological and ultrastructural observations are the most direct and classic diagnostic methods for FLHS. Our results showed that liver cells presented fatty pathological changes with abundant lipid droplets accumulating in the cytoplasm and the nucleus extruding to the cell edge in the FLHS model. However, the concentrations of HDL-Ch in the FLHS models were noted to first increase and then decrease compared with those the controls. Numerous studies revealed that HDL-Ch is the “favourable cholesterol” in the mammal body and is presented as a scavenger that removes excess cholesterol from the bloodstream^21,22^. Increased fat synthesis leads to an HDL-Ch compensatory increase in transported peripheral excess lipids to the liver during the early stage of FLHS progression. With the aggravation of liver fat deposition and liver injury, apolipoprotein synthesis (apoA, apoB, etc.) was impaired in the liver and then accelerated the occurrence of FLHS^5^. Overall, the HELP diet-induced FLHS model was well established according to our results.

Insulin is the only hypoglycaemic hormone in the body. It binds to cell surface-specific receptors to activate receptor substrates and then regulates the metabolism of glucose and lipids as well as the synthesis of proteins via downstream protein molecules. Insulin stimulates peripheral organs to take up glucose from plasma while inhibiting glucose output from the liver and fuels the conversion of glucose into glycogen or acetyl-CoA, which works as an intermediate in the tricarboxylic acid (TCA) cycle to facilitate the synthesis of fatty acids and amino acids^23,24^. When insulin resistance occurs in the body, insulin and its signalling pathway cannot be effectively activated and secreted in a compensatory manner, leading to the development of hyperinsulinaemia and hyperglycaemia^25^. Medical studies revealed that glucotoxicity and lipotoxicity exacerbate oxidative stress and promote the release of inflammatory factors, which contribute to impaired cellular functions^26,27^. Therefore, insulin resistance is expected to cause disordered metabolic homeostasis of glucose, lipids and proteins in the body and trigger oxidative stress and the release of pro-inflammatory factors as well as injury to the liver and other organs.

Fasting insulin and glucose levels are currently used as important indicators for determining the presence of insulin resistance. The results of the present study showed that blood glucose and insulin levels of layers in the HELP group were significantly higher than those of the control group, with signs of hyperinsulinaemia and hyperglycaemia resembling insulin resistance, and these were further confirmed by changes in serum inflammatory factors, including TNF-α and IL-6 in the HELP group^28–30^. To further determine the level of insulin resistance in the process of FLHS, an OGTT and IST were performed. The blood glucose level of the layers in the HELP group was significantly increased, and the clearance rate of glucose was decreased compared to that of the control group after oral administration of glucose in the OGTT. While the blood glucose level was significantly decreased by exogenous insulin in the layers and the clearance rate of glucose in the HELP group was lower than that of the control group in the IST, these results suggested that the insulin sensitivity of the FLHS layers was significantly diminished compared with the control layers.

It is generally recognized that the blockage of post receptor signal transduction of insulin is an essential mechanism of insulin resistance. The InsR/PI3K/AKT signalling pathway has been extensively studied as the main pathway for insulin-regulated energy metabolism^31,32^. Moreover, phosphorylation of various kinases (e.g., PI3K and AKT) in the insulin signalling pathway is critical for the normal physiological function of insulin^33^. Our results show that the expression of InsR mRNAs in the liver decreased in the FLHS model, suggesting that insulin resistance occurred during FLHS induction along with disturbed transduction of the InsR/PI3K/AKT signalling pathway. Multiple factors, such as oxidative stress and inflammation, could block the insulin signalling pathway^34,35^. In addition, the expression of TOR and S6K1, downstream effectors of the PI3K/AKT signalling pathway, decreased in the present study. TOR is considered a cellular nutrient sensor that can be stimulated by glucose and amino acids. Energy substance intake from the diet may induce the inhibition of AMPK activity, leading to increased TORc1 activity. Therefore, the phosphorylation of S6K1 is directly or indirectly regulated by cellular energy and nutrient levels. Recently, studies revealed that TORc1 and S6K1 may specifically reduce the phosphorylation level of AKT protein via negative feedback regulation, which further aggravates disorders of the Insulin/InsR/AKT signalling pathway^36^.

Forkhead box protein O1 (FOXO1) is a transcription factor negatively regulated by insulin signals. FOXO1 can be translocated from the nucleus to the cytoplasm via the AKT signalling pathway and participates in the regulation of hepatic gluconeogenesis and the synthesis of VLDL and fatty acids^37^. In addition, Srebp-1 is a pivotal gene involved in the expression of lipid synthesis-related genes in the liver. Studies have shown that the upregulated expression of Srebp-1c is closely related to NAFLD^38,39^. Wang *et al*. suggested that resveratrol can reduce the expression of Srebp-1c, a fat synthesis-related gene, by regulating the Sirt1-FOXO1 signalling pathway to inhibit the fatty pathological changes in cells^40^. In the present study, the expression of FOXO1 and Srebp-1 genes was significantly elevated in layers of the FLHS group.

In addition, glucose metabolism disturbance is an important indicator of insulin resistance. Insulin specifically binds to cell-surface insulin receptors (InsR) to activate AKT and subsequently enhances the synthesis of glycogens, the expression of glucose transporters in the tissue, and the translocation of Glut-4 to the cell membrane. However, studies found that Glut-4 expression was not detectable during, after hatching, or even in adulthood. Thomas *et al*. concluded that other glucose transporters instead of Glut-4 may exist in the birds for intracellular glucose transport^41^. Glut-1 is widely expressed in various tissues and cells and is the main protein for transporting glucose across the tissue barrier. Zhao *et al*. suggested that although Glut-1 protein was not sensitive to insulin stimulation in mammals, it was upregulated in chicken myoblasts treated with insulin^42^. Glut 3 and Glut-8 function in a way that is very similar to Glut-4 and are involved in cellular glucose transport^43^. For this purpose, the expression of transporters including Glut-1, Glut-3, and Glut-8 was determined by qPCR in our study, and the results showed that the expression of Glut-1 and Glut-3 in the liver of the HELP group was significantly reduced in the later stage of the experiment, suggesting decreased blood glucose intake in layers of the HELP group.

Metabolomics is currently one of the hotspots in biomedical research, and it is a powerful holistic and macroscopic approach for acquiring metabolic information in organisms or cells based on genomic and proteomic concepts^44–46^. In our study, insulin sensitivity was altered in the FLHS model induced by a HELP diet, and abnormal expression of genes related to the regulation of glucose and lipid metabolism was noted. The systematic understanding of the metabolism in the liver of the FLHS layers was insufficient; therefore, a non-targeted GC-MS/MS technique was applied in the present study to investigate the changes in small molecules in FLHS layers after the development of insulin resistance. The contents of glucose derivatives, such as L-glucan, 1,5-anhydroglucitol, tagatose, trehalose and sorbitol, were decreased in the livers of FLHS layers. This result suggested that oxidative decomposition of glucose was inhibited during FLHS development. The increased content of fumaric acid, an intermediate of the TCA cycle, in FLHS layers suggested that mitochondrial function was impaired in the FLHS layers. Previous studies revealed that the intermediate TCA cycle, such as fumaric acid, citrate, and succinic acid, was increased in diabetes^47^. Elevated carbohydrates such as hexadecanol, hexadecane and linoleic acid also revealed a high energy level in the liver of the FLHS model. In addition, the reduction in glycerine monostearate in our results indicated that TG synthesis in the liver was impaired. Once insulin resistance develops, the ability of TG lipolysis is enhanced, and a large amount of free fatty acids (FFAs) are released into the blood, further aggravating insulin resistance. Linoleic acid is an essential fatty acid in the body. Previous studies suggested that it has significant antioxidant effects, lowering lipids in serum and softening blood vessels in cardiovascular disease^48^. However, increasing clinical evidence has revealed that linoleic acid is increased in the serum of patients with insulin resistance, and other studies revealed that linoleic acid is elevated in the carbon tetrachloride-induced impaired liver^49,50^. The results of our study demonstrated that linoleic acid in the liver of FLHS layers was increased, suggesting that the liver is damaged in FLHS. Ascorbic acid is an important antioxidative substance in the body, and studies have shown that antioxidation substances such as ascorbic acid and galactitol are closely related to the onset of diabetes and ascites syndrome in broilers. Collectively, the decreased ascorbic acid content in FLHS layers indicated the presence of oxidative stress in the liver induced by high-energy low-protein diets and the development of insulin resistance in the FLHS process.

GO functional and pathway enrichment analysis of these metabolites with different contents in the liver of FLHS layers revealed disturbed amino acid metabolism, including the metabolism of phenylalanine, aspartic acid and glutamate; the biosynthesis of phenylalanine, tyrosine, and tryptophan; the metabolism of glutathione; and the biosynthesis of cysteine, histidine, valine, leucine, and isoleucine. Insulin is an important hormone regulating the metabolism of amino acids, and the synthesis of protein is disturbed when insulin deficiency or resistance occurs, leading to disturbed amino acid metabolism^51^. Current studies have shown that the disturbance in amino acid metabolism is closely related to the occurrence of various nutritional diseases, such as nonalcoholic fatty liver and diabetes^52,53^. Our study showed that non-essential amino acids such as alanine, glutamine and phenylpyruvate, were increased, while the essential amino acids such as valine, isoleucine, threonine and methionine were decreased in the liver of FLHS layers. The TCA cycle is the common metabolic pathway of the three major nutrients and is also a key link in energy metabolism in the organism. Metabolic disorder of the TCA cycle can further reflect that of amino acids in the body. Amino acids with different contents in the liver of FLHS layers belong to the glycogenic amino acid group, indicating a disturbance of glucose metabolism in the liver. Recently, studies have shown that branched amino acids, such as leucine and proline, are significantly higher in obese individuals than in individuals with normal weights^51,54^. Mukherjee *et al*. compared the amino acid metabolism profiles between alcoholic and nonalcoholic fatty liver disease patients and found significant differences in amino acid metabolism profiles in different liver diseases^55^. Along with the GS/MS results, high-energy low-protein diets resulted in an intake of excessive energy and inadequate amino acids during the process of FLHS as well as a disturbance in the conversion of amino acids to glucose in the layers through the TCA cycle, which further aggravated the amino acid deficiency in FLHS.

## Conclusion

For the first time, this study characterizes detection of the level of insulin resistance in FLHS induced by a HELP diet and provides clues suggesting that insulin resistance is highly involved in the occurrence of FLHS; comprehensively, metabolites participating in dysregulated biological processes and metabolism may serve as indicators for evaluating FLHS progression.

## Materials and Methods

### Ethics statement

All experimental protocols were approved by the Committee for the Care and Use of Experimental Animals of Jiangxi Agriculture University, and the ethics committee of Jiangxi Agriculture University specifically approved this study. Samples were collected in the case of animal anaesthesia with diethyl-ether pre-treatment.

### Animals

A total of 72 healthy, 90-day-old Hyline layers were obtained from a commercial layer hen farm. The layer hens were randomly divided into two groups (control group, HELP group), and each group had 36 layers with 6 replicates. For all treatments, the animals were acclimatized for 30 days at an animal farm under hygienic conditions prior to the commencement of the experiment. Layers in the formal experiment were 120 days old.

### Experimental protocol

The layers in the control group (36 layers) were fed a basal diet (metabolic energy 11.21 MJ/kg, crude protein 15.86%) formulated to meet the nutritional requirements of layers (NRC, 1998), while the layers in the HELP group were fed a high-energy low-protein diet (metabolic energy 12.97 MJ/kg, crude protein 12.00%), and the other nutritional ingredients were the same as those in the basal diet. The detailed feed formulation and the feed nutrition levels of each group are shown in Table 3. And all layers were maintained with ad libitum access to food and water with 16–17 h of light time during the formal experiment. The whole experimental period lasted 120 days.

**Table 3:** Coposition and nutrients levels of diets (air-dry basis) %.

Composition of diet %	Control Group	HELP group
Corn	64.00	70.00
Wheat bran	2.00	1.20
Soybean meal	24.00	14.58
fat-Soybean oil	0	4.22
Calcium	8.00	8.00
*Premix	2.00	2.00
Total	100.00	100.00
**Nutrient level**		
Crude Protein CP	15.86	12.00
Available Phosphorus (AP)	0.51	0.46
Arginine	1.03	0.74
Methionine	0.37	0.32
Valine	0.77	0.58
Metabolic energy (kcal/kg)	2678.99	3100.00
Met + Cys	0.67	0.56

*The ingredient of premix: The ingredient of premix: multiple vitamins, 30 mg; cupric sulfate, 4.6 mg; ferrous sulfate, 28.4 mg; manganous sulfate, 35.46 mg; zinc sulfate, 76 mg; zeolite powder, 6 mg; sodium selenite, 5 mg; anti-oxidizing quinolone, 50 mg; choline, 90 mg; bacitracin zinc, 26.7 mg; bran, 350 mg; methionine,100 mg.

### Sample collection and procedures

On experimental days 40, 80 and 120, twelve birds were randomly selected from the 6 replicates in each group, and then all layers were weighed after fasting for 12 h. Then, the 12 randomly selected layers from each group were anaesthetized and humanely sacrificed after blood collection, and the livers were removed. The living layers, livers, and abdominal fat pads were weighed, the indexes of liver (liver weight/live bird weight) and abdominal fat (abdominal fat pad/live bird weight) were calculated, and fat percent in the liver was determined by the Soxhlet extraction method^56^. The remaining livers were stored by different methods to observe related indexes.

### Serum biochemical indexes examination

Total triglyceride (TG), total cholesterol (T-Ch), high-density lipoprotein cholesterol (HDL-Ch), alanine aminotransferase (ALT) and aspartate aminotransferase (AST) levels in serum were determined according to the manufacturer’s instructions on experimental days 40, 80 and 120. Then, an ultraviolet and visible spectrophotometer (UV-754), semi-automatic biochemical analyser and enzyme standard instrument were used to determine their concentrations and activities. The commercial kits for these assays were purchased from Nanjing Jiancheng Bioengineering Institute (Nanjing, China).

### Histopathological and ultrastructural observation

On experimental day 120, the liver tissues from layers were dissected and either washed with cold saline and fixed in 10% neutral buffered formalin or fixed in OCT after freezing via gradient cooling in liquid nitrogen. The formalin-fixed samples and OCT-fixed samples were routinely processed, sectioned at 5 μm, and stained with haematoxylin and eosin (H&E) and Oil red O staining. The stained sections were observed using an optical microscope, and photographs were taken.

Transmission electron microscopy studies were performed as previously described^57^. The liver tissues were fixed in 2.5% neutral buffered glutaraldehyde, and the tissues were routinely processed and viewed under transmission electron microscopy (TEM) with a Zeiss 900 microscope (Zeiss, Germany).

### Insulin, IL-6, TNF-α and glucose concentration determination

Concentrations of insulin, IL-6 and TNF-α in serum from layers on experimental days 40, 80 and 120 were determined according to a commercial ELISA kit (CUSABIO, China) according to the manufacturer’s instructions. In addition, the concentrations of glucose in blood were determined using a handheld glucometer (ACCU-CHEK, US).

### Oral glucose tolerance test (OGTT)

The OGTT was determined as described in a previous study with a slight modification^58^. In brief, on experimental days 40, 80 and 120, layers in the control and HELP groups were fasted for 12 h, and six layers in each group were selected to receive a glucose bolus (2 g/kg BW, 20% w/v H_2_O) by oral gavage. Then, blood glucose concentration was determined at 0 min, 15 min, 30 min, 120 min, 240 min, and 300 min via small brachial blood vessels using a handheld glucometer (ACCU-CHEK, US). The AUC was calculated by GraphPad Prism7.

### Insulin sensitivity test (IST)

The IST was determined as previously described with a slight modification^58^. Briefly, on experimental days 40, 80 and 120, six layers in each group were selected for insulin treatment. Insulin-treated chickens received 100 µg/kg BW bovine insulin (Solarbio, China; diluted in 1X PBS) via i.p. injection, and then blood glucose concentration was determined at 0 min, 15 min, 30 min, 60 min, and 120 min. AUCs were calculated by GraphPad Prism7.

### Real-time quantitative polymerase chain reaction

The gene expression levels were assessed through real-time quantitative polymerase chain reaction (RT-qPCR). First, total RNA from the liver was isolated using TRIzol reagent (Takara, Japan) according to the manufacturer’s instructions, and the concentration of RNA was determined by a GeneQuant 1300 spectrophotometer. Reverse transcription was performed using a commercial kit (Takara, Japan) according to the manufacturer’s instructions. The specific primers for insulin receptor (InsR), TOR, S6K1, FOXO1, 4EBP1, GLUT-1, GLUT-3, GLUT-8 and SREBP-1 were designed using Primer Premier software (PREMIER Biosoft International, USA) and are shown in Table S2. The protocol used for amplification of the PCR products consisted of denaturation at 95 °C for 8 min, followed by 40 cycles of amplification at 95 °C for 10 s, then annealing and extension at 60 °C for 45 s. At the end of the PCR protocol, melting curve analyses were performed for all genes. All reactions were performed on an ABI Quant7.0 (Applied Biosystems, USA). Relative gene expression levels were calculated based on qPCR efficiency (E) and difference in the cycle threshold (ΔCt) of the treated versus control groups for both target and reference genes according to the formula. Ct represents the cycle threshold, ΔCt (sample) = Ct (target gene) − Ct (reference gene), ΔCt (calibrator) = Ct (target gene) − Ct (reference gene), ΔΔCt = ΔCt (sample) − ΔCt (calibrator), and 2-ΔΔCT represents the relative expression of the initial complementary DNA (cDNA) of the target gene. The internal reference β-actin or GAPDH gene was used as an internal control for the normalization of the results.

### Sample preparation for the metabolome analysis

The liver samples from layers were prepared for GC/MS-based metabolome analysis according to a previous study with a slight modification. In brief, ① each 50 mg liver sample was mixed with 0.4 mL of extraction mixed-solvent (V methanol: V chloroform = 3:1) and 20 µl of L-2-chlorophenylalanine (1 mg/mL stock in dH_2_O, Sigma-Aldrich, Shanghai, China) as an internal standard, mixed with a vortex for 30 s and homogenized in a ball mill for 6 min at 45 Hz. After the suspension had been centrifuged at 12000 rpm for 15 min at 4 °C, the supernatant (0.36 mL) was transferred into a fresh 2 mL GC/MS glass vial, and 18 μL supernatant from each sample was pooled as a QC sample. ② The resultant supernatant was lyophilized using a freeze dryer. The lyophilized samples were mixed with 20 mg/mL methoxyamine hydrochloride (Sigma-Aldrich, Shanghai, China) dissolved in pyridine and then incubated for 30 min at 80 °C. Next, the BSTFA reagent (1% TMCS, v/v) was added to the sample aliquots, and the mixture was incubated for 2 h at 70 °C. After incubation, the mixture was added to 8 µL FAMEs (standard mixture of fatty acid methyl esters, C8–C16: 1 mg/mL; C18–C24: 0.5 mg/mL in chloroform) and the QC sample, and the resultant supernatant was subjected to GC/MS analysis.

### GC/MS metabolite profiling of liver tissue

GC/TOF/MS analysis was performed using an Agilent 6890 gas chromatograph system coupled with a Pegasus HT time-of-flight mass spectrometer. The system utilized a DB-5MS capillary column coated with 5% diphenyl cross-linked with 95% dimethylpolysiloxane (30 m × 250 μm inner diameter, 0.25 μm film thickness; J&W Scientific, Folsom, CA, USA). A 1 μL aliquot of the analyte was injected in splitless mode. Helium was used as the carrier gas, the front inlet purge flow was 3 mL/min, and the gas flow rate through the column was 1 mL/min. The initial temperature was kept at 80 °C for 1 min, then raised to 295 °C at a rate of 10 °C/min, and then kept for 7 min at 295 °C. The injection, transfer line, and ion source temperatures were 280, 280, and 250 °C, respectively. The energy was −70 eV in electron impact mode. The mass spectrometry data were acquired in full-scan mode with an m/z range of 33–600 at a rate of 20 spectra per second after a solvent delay of 300 s.

### Processing and analysis of metabolite date

Chroma TOF4.3X software of LECO Corporation and LECO-Fiehn Rtx5 database were used for raw peak exacting, the data baseline filtering and calibration of the baseline, peak alignment, deconvolution analysis, peak identification and integration of the peak area [1]. The RI (retention time index) method was used in the peak identification, and the RI tolerance was 5000^59^.

Overall, 766 peaks were detected, and the internal standard normalization method was employed in this data analysis. The resulting three-dimensional data involving the peak number, sample name, and normalized peak area were fed to the SIMCA14 software package (Umetrics, Umea, Sweden) for principal component analysis (PCA) and orthogonal projections to latent structures-discriminate analysis (OPLS-DA)^60^. To obtain a higher level of group separation and obtain a better understanding of the variables responsible for classification, supervised orthogonal projections to latent structures-discriminant analysis (OPLS-DA) was applied. To refine this analysis, the first principal component of variable importance projection (VIP) was obtained, and the remaining variables were then assessed by Student’s t test (*P* > *0.05*)^61^; variables were discarded between two comparison groups. To better understand metabolite changes, functional and integrative analyses of upregulated or downregulated metabolite data *(P* < *0.05)* were performed in MetaboAnalyst 4.0 for enrichment and pathway analysis.

### Statistical analyses

The data were analysed using Prism 6.0 and SPSS version 17.0 (SPSS Inc., Chicago, IL, USA). Experiment data are shown as the means ± SD or SEM. Data between two groups were analysed by unpaired t test (Prism 7.0) if the data were in Gaussian distribution and had equal variance or by unpaired t test with Welch’s correction (Prism 7.0) if the data were in Gaussian distribution but with unequal variance. A *P* value less than 0.05 was considered statistically significant.

## Supplementary information

Supplementary Data
