# Supplementary material for: Insulin resistance and metabonomics analysis of fatty liver haemorrhagic syndrome in laying hens induced by a high-energy low-protein diet
Source: Sci Rep. 2019 Jul 12;9:10141. doi: 10.1038/s41598-019-46183-y (PMC6626135; doi:10.1038/s41598-019-46183-y)
Supplement: Supplementary file 1 — Supplementary Data [file 41598_2019_46183_MOESM1_ESM.docx]

**Supplemental materials:**

**SREP-18-40550A**

**Title: Insulin resistance and metabonomics analysis of fatty liver haemorrhagic syndrome in laying hens induced by a high-energy low-protein diet**

Yu Zhuang^＆^, Chenghong Xing^＆^, Huabin Cao，Caiying Zhang, Junrong Luo, Xiaoquan Guo, Guoliang Hu^*^

Jiangxi Provincial Key Laboratory for Animal Health, Institute of Animal Population Health, College of Animal Science and Technology, Jiangxi Agricultural University, No. 1101 Zhimin Avenue, Economic and Technological Development District, Nanchang 330045, Jiangxi, PR China

**&these two are equal first authors**

***Corresponding authors:** Guoliang Hu, Email: hgljx3818@163.com

**All authors have read the manuscript and agreed to submit it in its current form for consideration for publication in the journal.**

**Table S1 Model resolution analysed by PCA**

| **Model** | **Type** | **A** | **N** | **R^2^X(cum)** | **R^2^Y(cum)** | **Q^2^ (cum)** |
| --- | --- | --- | --- | --- | --- | --- |
| M1 | PCA-X | 2 | 26 | 0.241 | - | -0.0445 |
| M2 | OPLS-DA | 1+1+0 | 20 | 0.195 | 0.978 | 0.46 |

**Table S2 Sequence of Target genes primer**

| **Gene names** | **Pubmed N.O.** | **Sequence of primer** | **Length of production** |
| --- | --- | --- | --- |
| Insulin  receptor | AF111857 | CAAACGGTGACCAAGCCTCA  CATCCTGCCCATCAAACTCCG | 186 |
| TOR | XM_417614 | CCAGGATTCTTCGGACTA  CCATCACAAACCCTTATT | 249 |
| S6K1 | NM_001030721 | CATGATTTCCAAACGACCAGA  AGTAAACCAAACAAGCCCTCC | 134 |
| FOXO1 | NM_204328 | ATGCGACCTCTGGTAATA  AAGTGTAGGCAAATCGTC | 307 |
| 4EBP1 | XM_424384 | ACCAGGATTATTTATGACCG  TTCACCTACATTCGCTTTCT | 174 |
| GAPDH | NM_204305 | AGAACATCATCCCAGCGTCC  CGGCAGGTCAGGTCAACAAC | 133 |
| GLUT-1 | NM-205209 | TAGTACTGGAGCAGGTGGCAGA  CGGCACAAGAATGGATGAAA | 124 |
| GLUT-3 | NM-205511 | TCCCCAGAGCTTCTTACCTCAC  CAGCAAAAGCCAAGACATTCAC | 148 |
| GLUT-8 | NM-204375 | CCAAATGGGAACAACTCATCAA  GGGCAAAACCAGCAACAAA | 127 |
| β-Actin | NM_205518 | TGTGCTGTCCCTGTATGCCTCT  TGGTGGTGAAGCTGTAGCCTCT | 194 |
| SREBP-1 | [NM_204126.2](http://www.ncbi.nlm.nih.gov/entrez/viewer.fcgi?db=nucleotide&id=806638696) | CATTGGGTCACCGCTTCTTCGTG  CGTTGAGCAGCTGAAGGTACTCC | 237 |


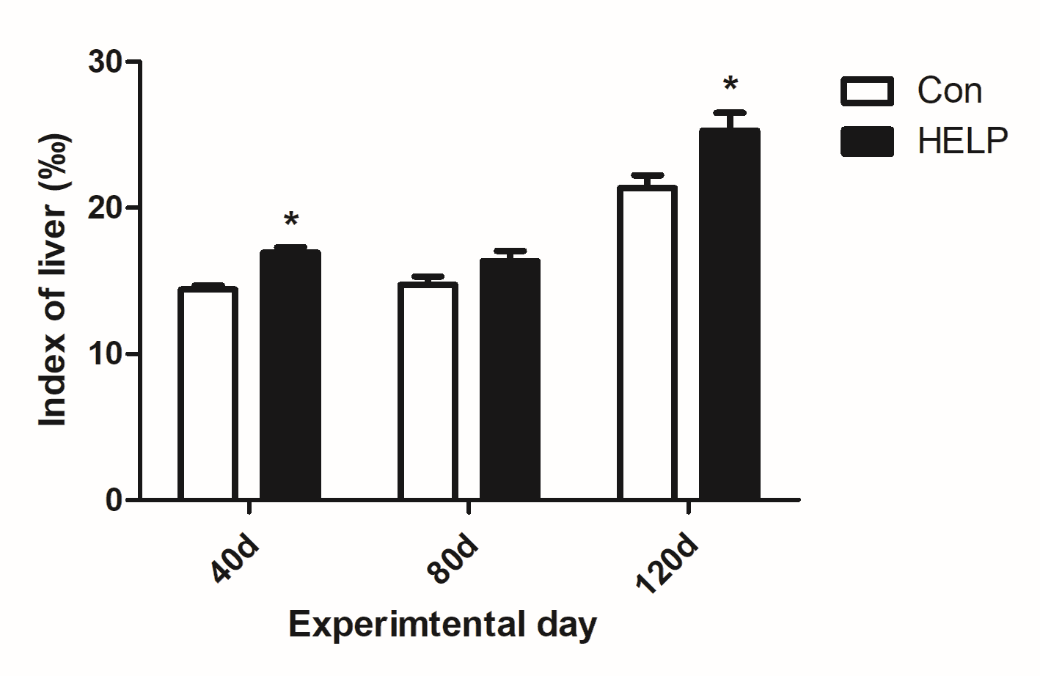


**Fig S1: Index of liver in both group during experimental period.** All values are means±SEM. Means with asterisks are significantly different from values of layer in control group.


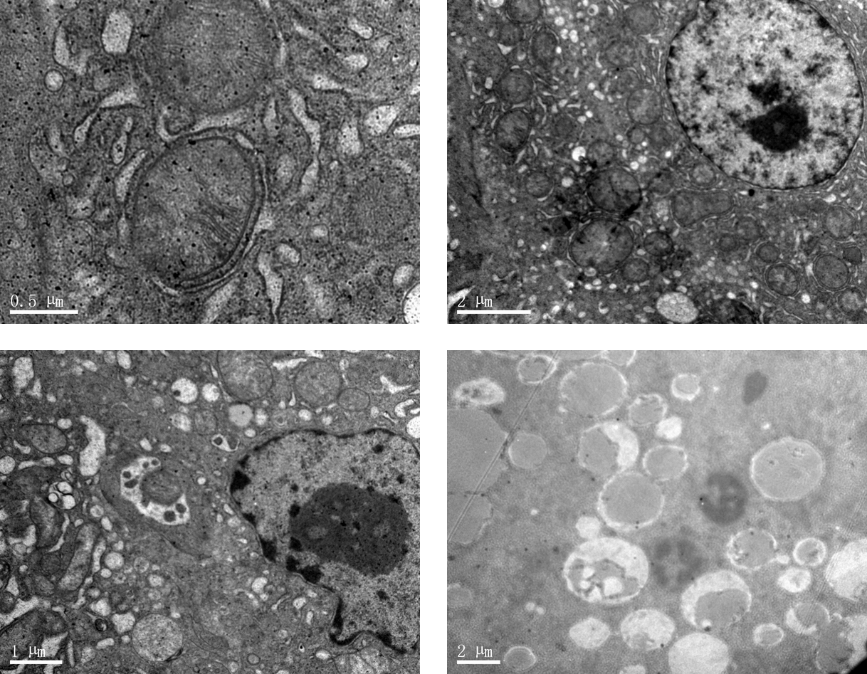


**Fig S2: Ultrastructural Observation in high energy-low protein (HELP) group (A and B) and Control group (C and D) in experimental day 120.**


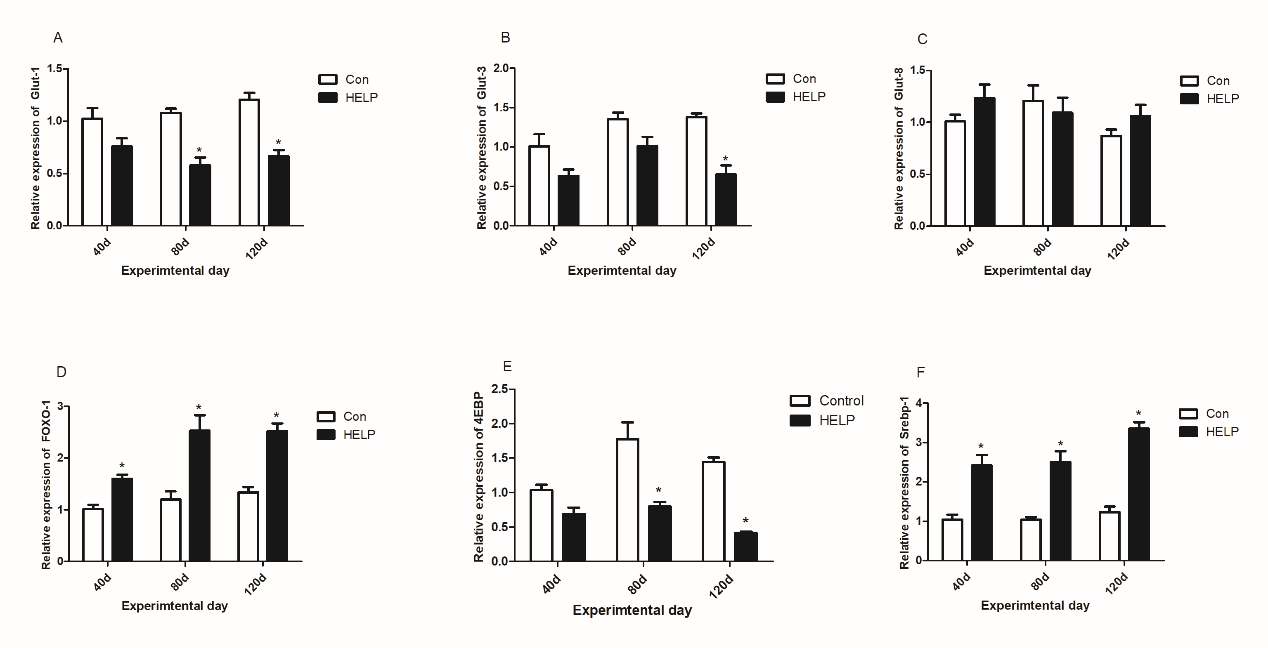


**Fig S3 Relative expression of downstream factors in insulin signal pathway.** The relative expression of Glut-1 (A), Glut-3(B), Glut-8 (C), FOXO1(D), 4EBP(E), Srebp-1(F) from liver in layers fed normal diet (control group) and HELP diet on day 40, 80, 120. All values are means ± SEM. Means with asterisks are significantly different from values of layer in control group.

**
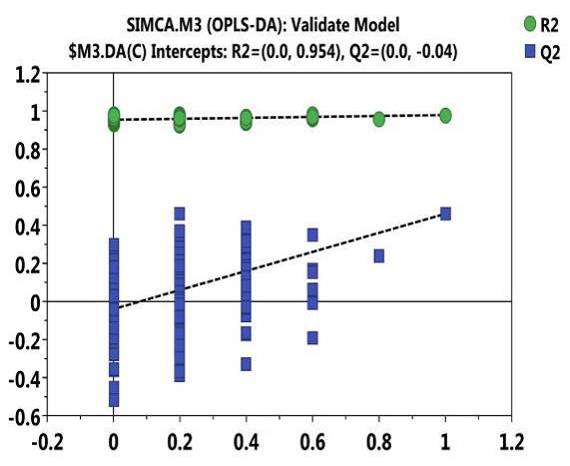
**

**Fig S4 Two hundred permutations were performed.** the resulting R2and Q2 values were plotted. Green circle: R2; blue square: Q2. The green line represents the regression line for R2 and the blue line for Q2.


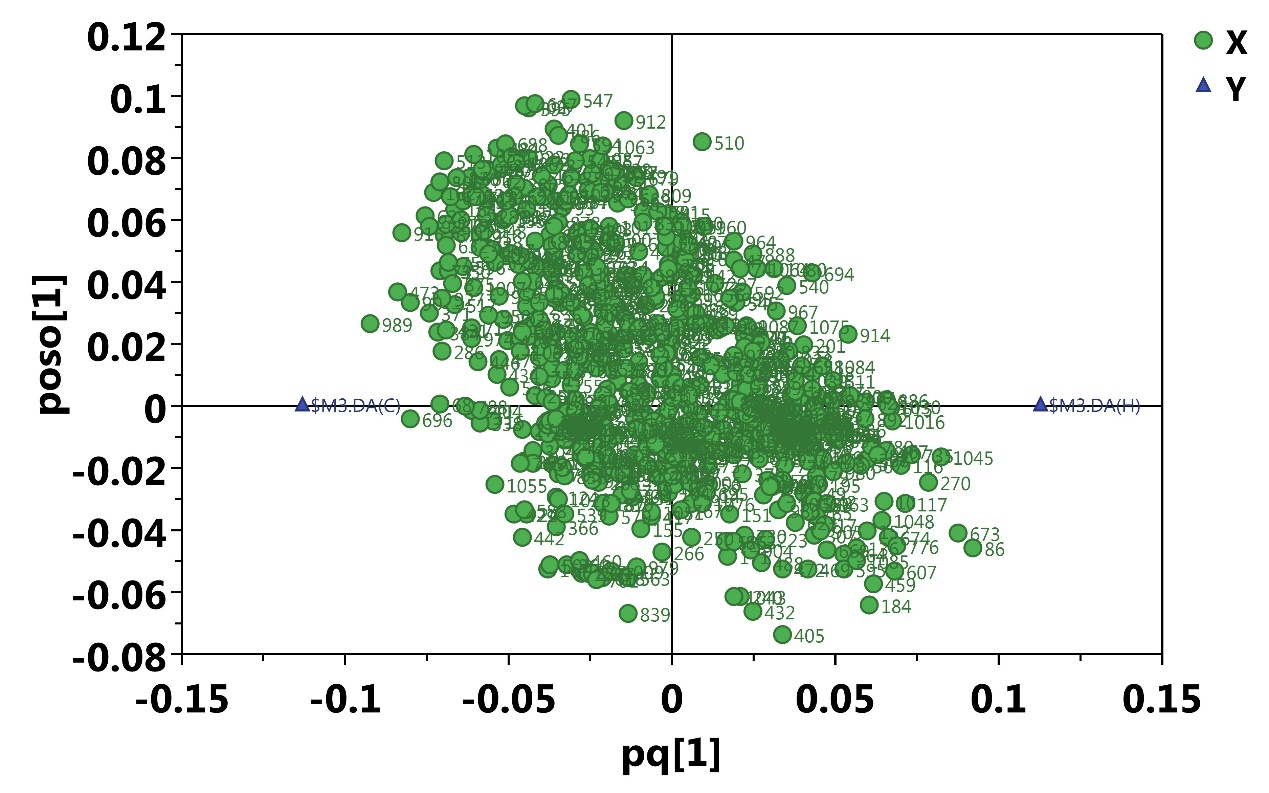


**Fig S5** Loading Scatter Plot of orthogonal partial least squares discrimination analysis (OPLS-DA )model in liver metabolites.
